# Exploring Potential Causal Genes for Uterine Leiomyomas: A Summary Data-Based Mendelian Randomization and FUMA Analysis

**DOI:** 10.3389/fgene.2022.890007

**Published:** 2022-07-12

**Authors:** Yuxin Dai, Xudong Liu, Yining Zhu, Su Mao, Jingyun Yang, Lan Zhu

**Affiliations:** ^1^ Department of Obstetrics and Gynecology, State Key Laboratory of Complex, Severe and Rare Diseases, National Clinical Research Center for Obstetric and Gynecologic Diseases, Peking Union Medical College Hospital, Chinese Academy of Medical Sciences and Peking Union Medical College, Beijing, China; ^2^ Medical Science Research Center, State Key Laboratory of Complex Severe and Rare Diseases, Peking Union Medical College Hospital, Chinese Academy of Medical Science and Peking Union Medical College, Beijing, China; ^3^ School of Mathematical Sciences, Fudan University, Shanghai, China; ^4^ Rush Alzheimer’s Disease Center, Rush University Medical Center, Chicago, IL, United States; ^5^ Department of Neurological Sciences, Rush University Medical Center, Chicago, IL, United States

**Keywords:** uterine leiomyomas, expression quantitative trait loci, summary Mendelian randomization, genome-wide association study, functional mapping

## Abstract

**Objective:** To explore potential causal genetic variants and genes underlying the pathogenesis of uterine leiomyomas (ULs).

**Methods:** We conducted the summary data-based Mendelian randomization (SMR) analyses and performed functional mapping and annotation using FUMA to examine genetic variants and genes that are potentially involved in the pathogenies of ULs. Both analyses used summarized data of a recent genome-wide association study (GWAS) on ULs, which has a total sample size of 244,324 (20,406 cases and 223,918 controls). We performed separate SMR analysis using CAGE and GTEx eQTL data.

**Results:** Using the CAGE eQTL data, our SMR analysis identified 13 probes tagging 10 unique genes that were pleiotropically/potentially causally associated with ULs, with the top three probes being ILMN_1675156 (tagging *CDC42*, PSMR = 8.03 × 10^−9^), ILMN_1705330 (tagging *CDC42*, PSMR = 1.02 × 10^−7^) and ILMN_2343048 (tagging *ABCB9*, PSMR = 9.37 × 10^−7^). Using GTEx eQTL data, our SMR analysis did not identify any significant genes after correction for multiple testing. FUMA analysis identified 106 independent SNPs, 24 genomic loci and 137 genes that are potentially involved in the pathogenesis of ULs, seven of which were also identified by the SMR analysis.

**Conclusions:** We identified many genetic variants, genes, and genomic loci that are potentially involved in the pathogenesis of ULs. More studies are needed to explore the exact underlying mechanisms in the etiology of ULs.

## Introduction

Uterine leiomyomas (ULs), also called myomas or uterine fibroids, are benign tumors in the smooth muscle tissue in myometrium (Bulun, 2013; Stewart et al., 2016). The overall prevalence of UL is about 70% in women of reproductive age, and approximately 25% of UL patients suffer from apparent clinical symptoms and require treatment (Stewart et al., 2017). ULs are the most prevalent benign tumor in female reproductive tract and the leading indication for hysterectomy. ULs represent a major cause of morbidity in women of childbearing age and account for excessive menstrual bleeding, pelvic pain or pressure, infertility, and pregnancy complications (Commandeur et al., 2015). To date, the only definitive treatment for ULs, including the familial subtype, is hysterectomy, which creates a great challenge if fertility preservation is desired. ULs also cause tremendous economic burden. For example, the annual cost of ULs in the US alone, including direct medical costs and indirect financial losses, is estimated to be up to $34.4 billion, higher than the combined cost of breast and colon cancer (Cardozo et al., 2012).

UL is a complex, multi-factorial gynecological benign disease with highly variable tumor size, tumor location and clinical manifestations. Many factors have been reported to be associated with the risk of ULs, including biological, demographic, reproductive and lifestyle factors (Parazzini, 2006; Sparic et al., 2016; Wise and Laughlin-Tommaso, 2016). Furthermore, previous studies also suggested that genetics plays an important role in the pathogenesis of ULs. For example, African-American women, or generally women with African origin, are more predisposed to develop ULs, with a prevalence as high as 80% (Day Baird et al., 2003), suggesting that ethnicity-specific factors, potentially ethnicity-specific genetic structure, may underlie the pathogenesis of ULs. Familial clustering between first-degree relatives and twins was also observed as well as multiple inherited syndromes in which fibroid development occurred (Luoto et al., 2000; Tomlinson et al., 2002). Moreover, many genome-wide association study (GWAS) and candidate gene studies have identified several genetic variants/loci associated with the susceptibility of ULs (Cha et al., 2011; Hellwege et al., 2017; Rafnar et al., 2018; Välimäki et al., 2018; Edwards et al., 2019; Gallagher et al., 2019). However, the role of putative risk factors and the underlying biological mechanisms underpinning ULs remain largely unclear, which has contributed to the slow progress in the development of effective treatment options for ULs. More studies are needed to explore genetic variants/genes that are potentially causally associated with ULs to better understand the pathogenesis of ULs.

Mendelian randomization (MR) uses genetic variants as the proxy to randomization. Recently, it has been widely adopted to explore pleiotropic/potentially causal effect of an exposure on various outcomes (e.g., ULs) (Davey Smith and Hemani, 2014). Confounding and reverse causation, which are commonly encountered in traditional association studies, can be greatly reduced by MR. This method has been successful in identifying gene expression probes or DNA methylation loci that are pleiotropically/potentially causally associated with various phenotypes, such as neuropathologies of Alzheimer’s disease and severity of COVID-19 (Liu et al., 2021a; Liu et al., 2021b).

In this paper, we attempted to prioritize genes that are potentially causally associated with ULs through a summary data-based MR (SMR) approach. We also performed functional mapping and annotation to further explore genetic variants and genomic loci that are potentially involved in the pathogenesis of ULs.

## Methods

### Genome-Wide Association Study Data for Uterine Leiomyomas

The GWAS summarized data for ULs were provided by a recent genome-wide association meta-analysis of ULs (Gallagher et al., 2019). The results were based on meta-analyses of ULs using data from four population-based cohorts (Women’s Genome Health Study, United Kingdom Biobank, Queensland Institute of Medical Research, and North Finnish Birth Cohort), with a total sample size of 244,324 (20,406 cases and 223,918 controls). Genotyping was done on different platforms, and imputation was performed using the reference panel from the 1000 Genomes Project European dataset (1000G EUR) Phase 3 or the Haplotype Reference Consortium (HRC) panel. For each cohort, logistic regression or linear mixed model association analysis was done, assuming an additive genetic model and adjusting for age, BMI, and/or the first five principal components, and/or array type, as appropriate. The GWAS summarized data can be downloaded at http://ftp.ebi.ac.uk/pub/databases/gwas/summary_statistics/GCST009001-GCST010000/GCST009158/.

### Expression Quantitative Trait Loci Data

The SMR analyses used cis-eQTL genetic variants as the instrumental variables (IVs) for gene expression. We performed separate SMR analysis using eQTL data from two sources. Specifically, we used the CAGE eQTL summarized data for whole blood (Lloyd-Jones et al., 2017), which included 2,765 participants, and the V7 release of the GTEx eQTL summarized data for uterus, which included 70 participants (Consortium, 2020). The eQTL data can be downloaded at https://cnsgenomics.com/data/SMR/#eQTLsummarydata.

### Summary Data-Based Mendelian Randomization Analysis

**FIGURE 1:**
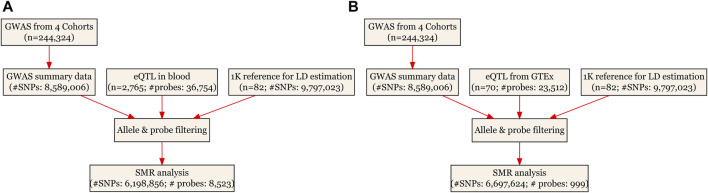
Flow chart for the SMR analyses. **(A)** SMR analysis using CAGE eQTL data from blood; and **(B)** SMR analysis using GTEx eQTL data. CAGE, Consortium for the Architecture of Gene Expression; eQTL, expression quantitative trait loci; GWAS, genome--wide association studies; GTEx, Genotype--Tissue Expression; LD, linkage disequilibrium; SMR, summary data--based Mendelian randomization; SNP, single nucleotide polymorphism.

The SMR analyses, based on the principle of MR (Davey Smith and Ebrahim, 2003), used cis-eQTL as the IV, gene expression as the exposure and ULs as the outcome. It was essentially a two-sample MR analysis which jointly analyzed summarized GWAS data and eQTL data from different samples to test for pleiotropic association between gene expression and a ULs. Detailed information regarding the SMR method was reported elsewhere (Zhu et al., 2016). The analyses were done using the software SMR, and we followed an approach similar to the one adopted in our previous publication (Liu et al., 2021b). The existence of linkage in the observed association was assessed using the heterogeneity in dependent instruments (HEIDI) test. P_HEIDI_<0.05 means rejection of the null hypothesis. That is, the observed association could be due to two distinct genetic variants in high linkage disequilibrium with each other. We adopted the default settings in SMR [e.g., P_eQTL_ <5 × 10^−8^ and minor allele frequency (MAF) > 0.01] and used false discovery rate (FDR) to adjust for multiple testing (for full details of the default settings, see Supplementary Table S1). The SMR analytic process is illustrated in Figure 1.

### FUMA Analysis

To better understand the genetic mechanisms underlying ULs, we also conducted a FUMA analysis to functionally map and annotate the genetic association, again using the GWAS summarized results of ULs. FUMA is an on-line platform that integrates information from multiple resources for easy implementation of post-GWAS analysis, such as functional annotation and gene prioritization (Watanabe et al., 2017). It has two processes, SNP2GENE, which annotates SNPs regarding their biological functions and maps them to genes, and GENE2FUNC, which annotates the mapped genes in biological contexts. In SNP2GENE, we performed both positional mapping and eQTL mapping using GTEx v8 of whole blood and uterus. We selected all types of genes in gene prioritization and adopted the default settings otherwise (e.g., maximum *p*-value of lead SNPs being 5 × 10^−8^ and r^2^ threshold for independent significant SNPs being 0.6). In GENE2FUNC, we adopted the default settings (e.g., using FDR to correct for multiple testing in the gene-set enrichment analysis).

Data cleaning and statistical/bioinformatical analysis was performed using R version 4.1.2 (https://www.r-project.org/), SMR (https://cnsgenomics.com/software/smr/), and FUMA (https://fuma.ctglab.nl/).

## Results

### Basic Information of the Summarized Data

In the SMR analyses, the CAGE eQTL has a much larger number of participants than that of the GTEx eQTL data (2,765 vs. 70), so is the number of eligible probes (8,523 vs. 999). After checking allele frequencies among the datasets and LD pruning, there were more than six million eligible SNPs in each SMR analysis. In the FUMA analysis, about 8.6 million SNPs were used as the input. The detailed information was shown in Table 1.

**TABLE 1:** Basic information of the eQTL and GWAS data.

Data Source	Total number of participants	Number of eligible genetic variants or probes
eQTL data		
CAGE	2,765	8,523
GTEx	70	999
GWAS data for SMR analysis		
WGHS	3,375/9,465	—
NFBC	363/5,000	—
QIMR	1,484/3,701	—
UKBB	15,184/205,752	—
Total	20,406/223,918	CAGE: 6,198,856; GTEx: 6,697,624
GWAS data for FUMA analysis	20,406/223,918	8,589,006

CAGE, Consortium for the Architecture of Gene Expression; eQTL, expression quantitative trait loci; GTEx, Genotype--Tissue Expression; GWAS: genome--wide association studies; WGHS, Women’s Genome Health Study; QIMR, Queensland Institute of Medical Research; UKBB, United Kingdom, Biobank; NFBC, North Finnish Birth Cohort.

### Pleiotropic Association With UL

**FIGURE 2:**
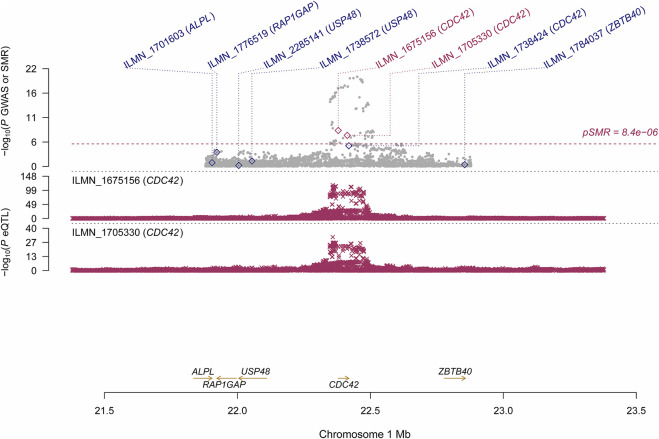
Pleiotropic association of *CDC42* with ULs using CAGE eQTL. Top plot, grey dots represent the --log10(*p* values) for SNPs from the GWAS of ULs, with solid rhombuses indicating that the probes pass HEIDI test. Middle plot, eQTL results. Bottom plot, location of genes tagged by the probes. CAGE, Consortium for the Architecture of Gene Expression; eQTL, expression quantitative trait loci; GWAS, genome--wide association studies; GTEx, Genotype--Tissue Expression; HEIDI, heterogeneity in dependent instruments; SMR, summary data--based Mendelian randomization; SNP, single nucleotide polymorphism; ULs, uterine leiomyomas.

**FIGURE 3:**
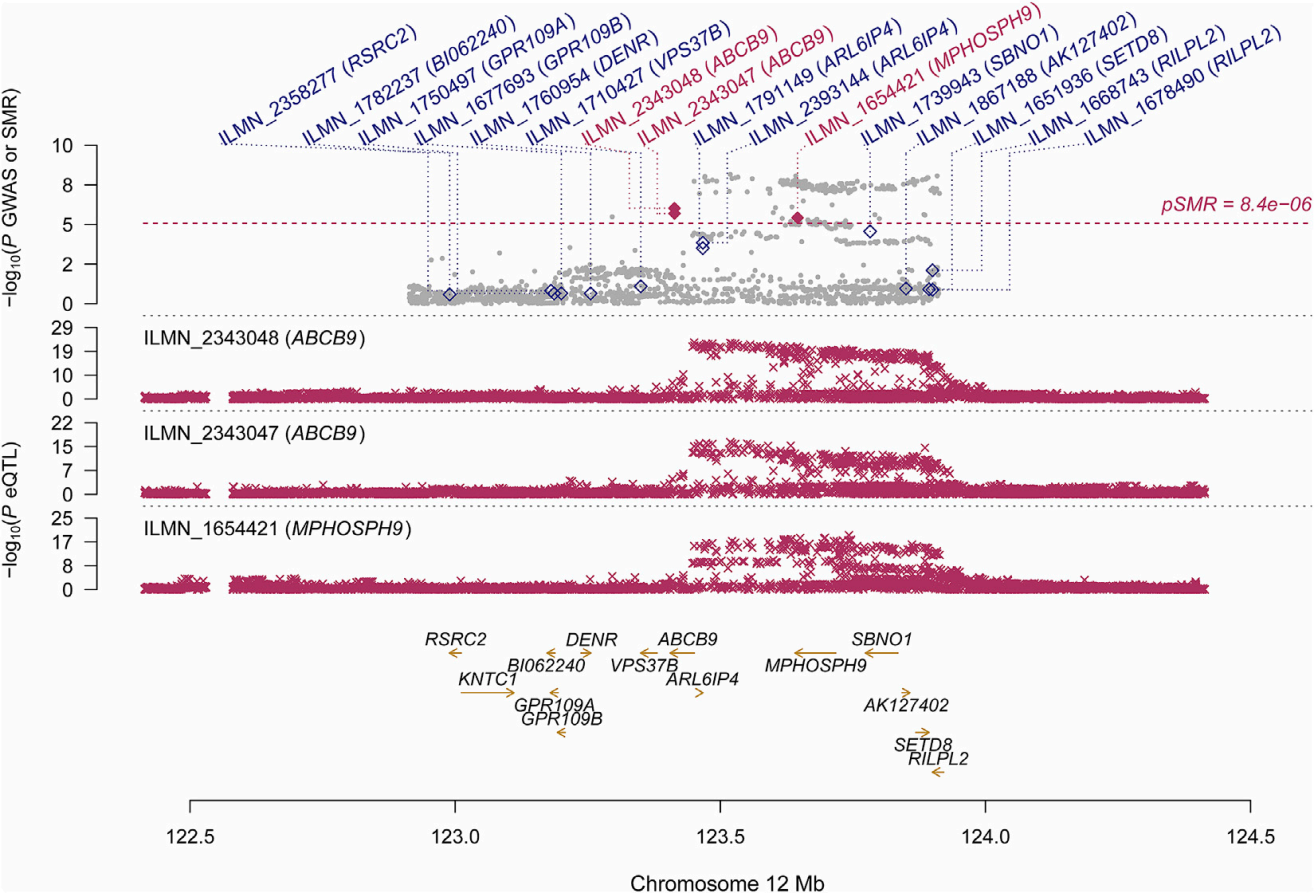
Pleiotropic association of *ABCB9* and *MPHOSPH9* with ULs using CAGE eQTL data. Top plot, grey dots represent the log10(p values) for SNPs from the GWAS of ULs, with solid rhombuses indicating that the probes pass HEIDI test. Middle plot, eQTL results. Bottom plot, location of genes tagged by the probes. CAGE, Consortium for the Architecture of Gene Expression; eQTL, expression quantitative trait loci; GWAS, genome--wide association studies; GTEx, Genotype--Tissue Expression; HEIDI, heterogeneity in dependent instruments; SMR, summary data--based Mendelian randomization; SNP, single nucleotide polymorphism; ULs, uterine leiomyomas.

Using the CAGE eQTL data, our SMR analysis identified 13 probes tagging 10 unique genes that were pleiotropically/potentially causally associated with ULs, with the top three probes being ILMN_1675156 (tagging *CDC42*, P_SMR_ = 8.03 × 10^−9^), ILMN_1705330 (tagging *CDC42*, P_SMR_ = 1.02 × 10^−7^) and ILMN_2343048 (tagging ABCB9, P_SMR_ = 9.37 × 10^−7^; Table 2). There were three probes tagging *CDC42* (Figure 2) and two probes tagging *ABCB9* (Figure 3) that showed significant pleiotropic association with ULs. Using GTEx eQTL data, we did not identify any genes that were pleiotropically/potentially causally associated with ULs after correction for multiple testing (Table 2).

**TABLE 2:** The top hit probes identified in SMR analysis*.

eQTL data	Probe	Gene	CHR	Top SNP	P_eQTL_	P_GWAS_	Beta	SE	P_SMR_	P_HEIDI_	Q value
Uterus	ENSG00000198496.6	*NBR2*	17	rs2292595	4.34 × 10^−27^	0.0002	0.0359	0.0100	0.0003	0.1417	0.2211
	ENSG00000155393.8	*HEATR3*	16	rs11642695	3.78 × 10^−12^	4.66 × 10^−5^	0.0871	0.0248	0.0004	0.5173	0.2211
	ENSG00000101751.6	*POLI*	18	rs4940321	1.31 × 10^−8^	5.47 × 10^−5^	−0.0960	0.0291	0.0010	0.4576	0.3252
	ENSG00000164535.10	*DAGLB*	7	rs13235365	5.58 × 10^−13^	0.0004	0.0404	0.0126	0.0014	0.9852	0.3505
	ENSG00000226752.3	*PSMD5--AS1*	9	rs4837796	3.37 × 10^−28^	0.0031	0.0265	0.0093	0.0043	0.8854	0.7269
	ENSG00000164048.9	*ZNF589*	3	rs11718329	2.06 × 10^−10^	0.0019	0.0419	0.0150	0.0051	0.3164	0.7269
	ENSG00000164045.7	*CDC25A*	3	rs4511915	3.80 × 10^−13^	0.0035	0.0314	0.0115	0.0065	0.1342	0.7269
	ENSG00000188878.12	*FBF1*	17	rs9674908	1.20 × 10^−8^	0.0022	−0.0578	0.0213	0.0068	0.6307	0.7269
	ENSG00000027001.7	*MIPEP*	13	rs75783226	1.95 × 10^−9^	0.0025	0.0498	0.0185	0.0070	0.1324	0.7269
	ENSG00000229759.1	*MRPS18AP1*	3	rs11130163	7.36 × 10^−15^	0.0052	0.0260	0.0099	0.0085	0.6590	0.7269
Whole blood	ILMN_1675156	*CDC42*	1	rs2473290	7.00 × 10^−118^	2.82 × 10^−9^	0.0896	0.0155	8.03 × 10^−9^	1.56 × 10^−8^	**6.84 × 10** ^−**5**^
	ILMN_1705330	*CDC42*	1	rs2473290	2.08 × 10^−32^	2.82 × 10^−9^	0.1778	0.0334	1.02 × 10^−7^	3.83 × 10^−5^	**0.0004**
	ILMN_2343048	*ABCB9*	12	rs4148856	1.17 × 10^−23^	1.96 × 10^−8^	−0.2064	0.0421	9.37 × 10^−7^	0.3185	**0.0027**
	ILMN_2343047	*ABCB9*	12	rs641760	9.79 × 10^−17^	7.64 × 10^−9^	−0.2509	0.0528	2.01 × 10^−6^	0.1106	**0.0043**
	ILMN_1654421	*MPHOSPH9*	12	rs10772996	7.72 × 10^−19^	6.34 × 10^−8^	−0.2501	0.0541	3.73 × 10^−6^	0.9425	**0.0064**
	ILMN_1767642	*C11orf46*	11	rs12364889	1.81 × 10^−34^	8.32 × 10^−7^	0.1410	0.0309	5.10 × 10^−6^	0.0977	**0.0072**
	ILMN_2266948	*SLC38A1*	12	rs11183420	5.41 × 10^−36^	2.33 × 10^−6^	0.1401	0.0316	9.36 × 10^−6^	0.0048	**0.0107**
	ILMN_1691188	*UIMC1*	5	rs353491	4.49 × 10^−17^	2.28 × 10^−7^	0.2164	0.0490	1.00 × 10^−5^	0.1816	**0.0107**
	ILMN_2359907	*CD68*	17	rs56319762	2.76 × 10^−51^	5.07 × 10^−6^	0.1093	0.0250	1.26 × 10^−5^	0.0157	**0.0119**
	ILMN_1654552	*MRPS31*	13	rs7324090	5.69 × 10^−10^	1.44 × 10^−9^	−0.3700	0.0853	1.44 × 10^−5^	0.3289	**0.0122**
	ILMN_1706531	*ABCC5*	3	rs4074672	3.24 × 10^−169^	1.61 × 10^−5^	0.0552	0.0129	1.94 × 10^−5^	0.2197	**0.0150**
	ILMN_1738424	*CDC42*	1	rs2038106	7.64 × 10^−30^	4.44 × 10^−6^	0.1430	0.0337	2.21 × 10^−5^	1.23 × 10^−9^	**0.0157**
	ILMN_1739943	*SBNO1*	12	rs1569068	1.30 × 10^−10^	2.92 × 10^−8^	0.3387	0.0807	2.73 × 10^−5^	0.8568	**0.0179**

* We showed the top ten pleiotropic association for the SMR analysis using GTEx eQTL data, and all the significant pleiotropic associations (after correction of multiple testing using FDR) in the SMR analysis using CAGE eQTL data. The GWAS, summarized data were provided by the study of Gallagher et al. and can be downloaded at http://ftp.ebi.ac.uk/pub/databases/gwas/summary_statistics/GCST009001-GCST010000/GCST009158/. The CAGE and GTEx eQTL data can be downloaded at https://cnsgenomics.com/data/SMR/#eQTLsummarydata.

P_eQTL_ is the p-value of the top associated cis-eQTL in the eQTL analysis; P_GWAS_ is the p-value for the top associated cis-eQTL in the GWAS analysis; Beta is the estimated effect size in SMR analysis; SE is the corresponding standard error; PSMR is the p-value for SMR analysis and P_HEIDI_ is the p-value for the HEIDI test.

Bold font means statistical significance after correction for multiple testing using FDR.

CAGE, Consortium for the Architecture of Gene Expression; CHR, chromosome; eQTL, expression quantitative trait loci; GTEx, Genotype--Tissue Expression; HEIDI, heterogeneity in dependent instruments; SNP, single--nucleotide polymorphism; SMR, summary data--based Mendelian randomization; FDR, false discovery rate; GWAS, genome--wide association studies.

### Functional Mapping and Annotation

**FIGURE 4:**
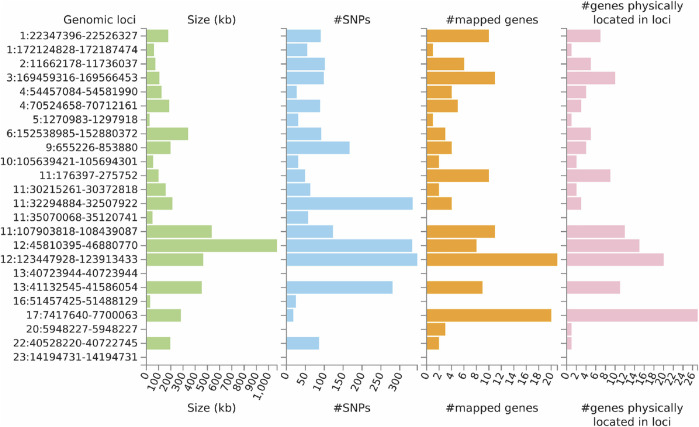
Genetic risk loci identified by FUMA analysis using GWAS data on ULs. Genomic risk loci are displayed in the format of “chromosome:start position--end position” on the Y axis. For each genomic locus, histograms from left to right depict the size, the number of candidate SNPs, the number of mapped genes (using positional mapping and eQTL mapping), and the number of genes known to be located within the genomic locus, respectively. CAGE, Consortium for the Architecture of Gene Expression; eQTL, expression quantitative trait loci; GWAS, genome--wide association studies; GTEx, Genotype--Tissue Expression; SNP, single nucleotide polymorphism; ULs, uterine leiomyomas.

FUMA analysis identified 106 independent significant SNPs, 33 lead SNPs (Supplementary Tables S2–S4), and 24 genomic risk loci (Figure 4; Supplementary Table S5). In addition, FUMA identified 137 genes that were potentially involved in the pathogenesis of ULs (Supplementary Table S6). These 137 genes are distributed in 20 genomic risk loci, with four genomic risk loci containing no identified genes (Figure 4 and Supplementary Table S6). Of the 137 identified genes, 7 were also identified by SMR analysis, including *CDC42*, *SLC38A1*, *ABCB9*, *MPHOSPH9*, *SBNO1*, *MRPS31* and *CD68*. Expression of the prioritized genes in 30 tissues can be found in Supplementary Table S7 and Supplementary Figure S1.

Gene-set enrichment analysis (GSEA) was undertaken to test the possible biological mechanisms of the 137 candidate genes implicated in ULs (Supplementary Table S8). A total of 96 gene sets with an adjusted *p* < 0.05 were identified. We found strong enrichment signals related with uterine fibroids (adjusted *p* = 2.00 × 10^−51^). In addition, we also found enrichment of sex-related signals such as GO_REGULATION_OF_GONAD_DEVELOPMENT (adjusted *p* = 0.023), endometriosis (adjusted *p* = 7.3 × 10^−4^), sex hormone-binding globulin levels (adjusted *p* = 0.003), and sex hormone levels (adjusted *p* = 0.024; Supplementary Table S8).

## Discussion

In this study, we conducted SMR and FUMA analysis to prioritize SNPs and genes to better understand the genetic mechanisms underlying ULs. We identified multiple genetic variants, genes, genomic risk loci and gene sets that may be involved in the pathogenesis of ULs. These findings provided helpful leads to a better understanding of the pathogenesis of ULs and highlight potential therapeutic targets for the treatment of ULs.

Several probes tagging *CDC42* (cell division control protein 42 homolog) showed significant pleiotropic association with ULs in the SMR analysis using CAGE eQTL data (Table 2). This gene was also identified by the FUMA analysis. *CDC42* is a member of the Rho family and is implicated in a variety of cellular functions including cell cycle progression, survival, transcription, actin cytoskeleton organization, and membrane trafficking (Huang et al., 2019). *CDC42* has been linked to multiple human cancers and is involved in the initiation of many cellular responses during oncogenic processes, such as transition from epithelial to mesenchymal, cell-cycle progression, migration/invasion, tumor growth, angiogenesis, and oncogenic transformation (Qadir et al., 2015; Maldonado and Dharmawardhane, 2018). Several studies reported that *CDC42* might also play an essential role in the pathogenesis of fibroid. For example, genome-wide analysis revealed that the 1p36.12 region, where *CDC42*/*WNT4* is located, was associated with uterine fibroids (Rafnar et al., 2018; Edwards et al., 2019; Gallagher et al., 2019). Interestingly, the genetic variant rs10917151 in *CDC42*/*WNT4* seems to have ancestry-specific effect on the risk of uterine fibroids. Specifically, the A allele was associated with a reduced risk of uterine fibroids in women of African ancestry (OR = 0.84) and an increased risk in women of European ancestry (OR = 1.16) (Edwards et al., 2019). Since rs10917151 has been reported to be involved in hormone-related traits (e.g., endometriosis and endometrial cancer), it probably plays a role in the development of leiomyomas *via* influencing hormone metabolism (Rafnar et al., 2018). Meanwhile, another study showed that the deregulation of *CDC42* influences fibroblasts activation which is essential in the pathogenesis of ULs (Zheng et al., 2014). Given the fact that increased cellular proliferation is present in fibroid compared with the adjacent uterine tissue and the function of *CDC42* in influencing cell cycle, further investigation is needed to elucidate the role of *CDC42* in the development of leiomyoma and the potential of this gene as a promising target for the prevention and treatment of ULs.

We also found that two probes tagging *ABCB9* (ATP binding cassette subfamily B member 9) showed significant pleotropic association with ULs in the SMR analysis using CAGE eQTL data. This gene was also identified in the FUMA analysis. *ABCB9* belongs to the superfamily of ATP-binding cassette (ABC) transporters which fulfill diverse physiological functions in different cellular localizations ranging from plasma membrane to intracellular membranous compartments (Linton, 2007). *ABCB9*, located on 12q24.31, is an antigen processing-like (TAPL) transporter that has been found to be involved in the development and progression of various malignant tumors, such as ovarian cancer and non-small cell lung cancer (Dong et al., 2014; Ween et al., 2015). The genetic variant rs2270788 in *ABCB9* was found to be associated with both the risk and tumor size of ULs in African American participants (Aissani et al., 2015). *ABCB9* was downregulated in women with a high level of progesterone serum (>1.5 ng/ml), compared with women with a lower level of progesterone serum (<1.5 ng/ml) (Labarta et al., 2011). Since progesterone is a major promoter of leiomyoma development and growth (Kim and Sefton, 2012), the role of *ABCB9* in fibroids in general, and its function in progesterone-driven growth of leiomyomas in particular, needs further exploration.

A recent GWAS study on ULs also performed two-sample Mendelian randomization analysis (Gallagher et al., 2019). However, the MR analysis was different from our SMR analyses in that their objective was to examine the causality of genetic association between UL and heavy menstrual bleeding (HMB). Their study identified 29 independent loci for ULs, with 27 of them on the autosomal chromosomes while our FUMA analysis identified a total of 24 genomic loci on the autosomal chromosomes. The definition of genomic locus was different between their approach and FUMA: their genomic locus was defined as regions of the genome containing all SNPs in LD (r^2^ > 0.6) with the index SNPs (independent SNPs, i.e., SNPs in low LD (r^2^ < 0.1) with nearby (≤500 kb) significantly associated SNPs), with any adjacent regions within 250 kb of one another being combined and classified as a single locus. In FUMA, independent significant SNPs (*p* < 5 × 10^−8^ and independent from each other at r^2^ < 0.6) were first identified. Then, all known SNPs in LD (r^2^ > 0.6) with one of the independent SNPs were included, using the pre-calculated LD structure based on 1000G. As a results, SNPs that were not originally in the GWAS results could also be included. This may partly explain the difference in the findings.

Findings from our SMR and FUMA analyses are not consistent, although we did identify several genes that were significant in both the SMR and FUMA analyses (Table 2 and Supplementary Table S3). This is likely due to the different focuses of the two types of analyses. The SMR analyses attempted to identify genes whose expressions were causally associated with ULs by using genetic variants as the instrumental variables (Zhu et al., 2016). By contrast, the FUMA analysis focused on genetic variants to characterize and annotate significant SNPs which were then mapped to genes for subsequent functional annotation by integrating knowledge from multiple resources (Watanabe et al., 2017). In one word, SMR focused on potentially causal association of gene expression, while FUMA attempted to pinpoint the most likely causal genetic variants and genes without differentiating whether the causal function is through gene expression or not.

There are three core assumptions underlying the SMR analyses: 1) the genotype is associated with gene expression; 2) the genotype is not associated with confounding factors that bias the associations between gene expression and ULs; and 3) the genotype is related to ULs only *via* its association with gene expression. Concern is minimal, moderate or cannot be directly verified for these assumptions. Specifically, for assumption 1, the SMR analyses adopted a *p*-value threshold of 5 × 10^−8^ to select the top associated eQTL. Therefore, we believe that the genetic variants we selected indeed showed strong association with gene expression and the concern of weak instrument is minimal. Assumption 2 is often based on biological belief that the genotype will not be associated with socioeconomic and behavioral characteristics that commonly confound the effects of exposure (i.e., gene expression) on the outcome (i.e., ULs) (Lawlor et al., 2008). This assumption could not be verified directly because we used summarized data for the SMR analyses. Violation of assumption 3 (i.e., horizontal pleiotropy) may distort MR results. It was found that horizontal pleiotropy can be detected in over 48% significant causal relationships in MR and introduced distortions as high as 201% in the causal estimates in MR. It can induce false-positive causal relationships in up to 10% of relationships (Verbanck et al., 2018). We did observe that the HEIDI test was significant for some of the observed associations, implying the existence of horizontal pleiotropy (Table 2). Caution should be exerted in interpretation of the findings in the presence of pleiotropy.

Our study has limitations. Although we identified multiple genes, such as *CDC42* and *ABCB9*, in pleiotropic association with ULs, we cannot directly compare expression of the identified genes between ULs patients and the normal control due to a lack of relevant gene expression data. Future studies are needed to examine changes in gene expression of the identified genes and further explore the possible pathogenic mechanisms. The incidence of ULs varies among ethnicities, implying the existence of ethnic-specific genetic architecture. However, the GWAS summarized data used in our analyses were based on participants of European ancestry. Moreover, ethnicity-specific gene expression and eQTL data are unavailable. As such, our findings might not be generalized to other ethnicities, and more studies comparing expression of the identified genes among different ethnicities are warranted. Previous research demonstrated that the SMR approach had good performance with a sample size of 1,000 and 10,000 for QTL summarized data and GWAS summarized data, respectively (Zhu et al., 2016). Therefore, power should not be a big concern for the SMR analysis using CAGE eQTL data which utilized eQTL data from 2,765 subjects and GWAS summarized data from 244,324 subjects. However, the SMR analysis using GTEx eQTL data might suffer from insufficient power with eQTL data from only 70 subjects. The number of eligible probes used in the SMR analyses was limited, especially in the analysis using GTEx eQTL (eligible probes = 999). We further checked and found that there were only 482 probes that were common in both GTEx eQTL data and the CAGE eQTL data, and none of the significant probes identified by using the CAGE eQTL data were included in the eligible probes in the GTEx eQTL data. Taken together, we believe that limited sample size and eligible probes was the major reason of null findings of the SMR analysis using GTEx eQTL data, and that we could not rule out the possibility of missing some important genes that were not tagged in the eQTL data. In addition, the FDR approach to correct for multiple testing resulted in additional possibilities of missing important genes.

## Conclusion

We identified many genetic variants, genes and genomic loci that are potentially involved in the pathogenesis of ULs. Biological validation of some of the identified genes is highly needed in future research, especially of the seven genes that were identified by both the SMR analysis using CAGE eQTL data and the FUMA analysis.

## Data Availability

All data generated or analyzed during this study are publicly available as specified in the methods section of this paper. Specifically, the eQTL data can be downloaded at https://cnsgenomics.com/data/SMR/#eQTLsummarydata, and the GWAS summarized data can be downloaded at http://ftp.ebi.ac.uk/pub/databases/gwas/summary_statistics/GCST009001-GCST010000/GCST009158/.
