# Supplementary figures and images for: Exploring Potential Causal Genes for Uterine Leiomyomas: A Summary Data-Based Mendelian Randomization and FUMA Analysis
Source: Front Genet. 2022 Jul 12;13:890007. doi: 10.3389/fgene.2022.890007 (PMC9315954; doi:10.3389/fgene.2022.890007)

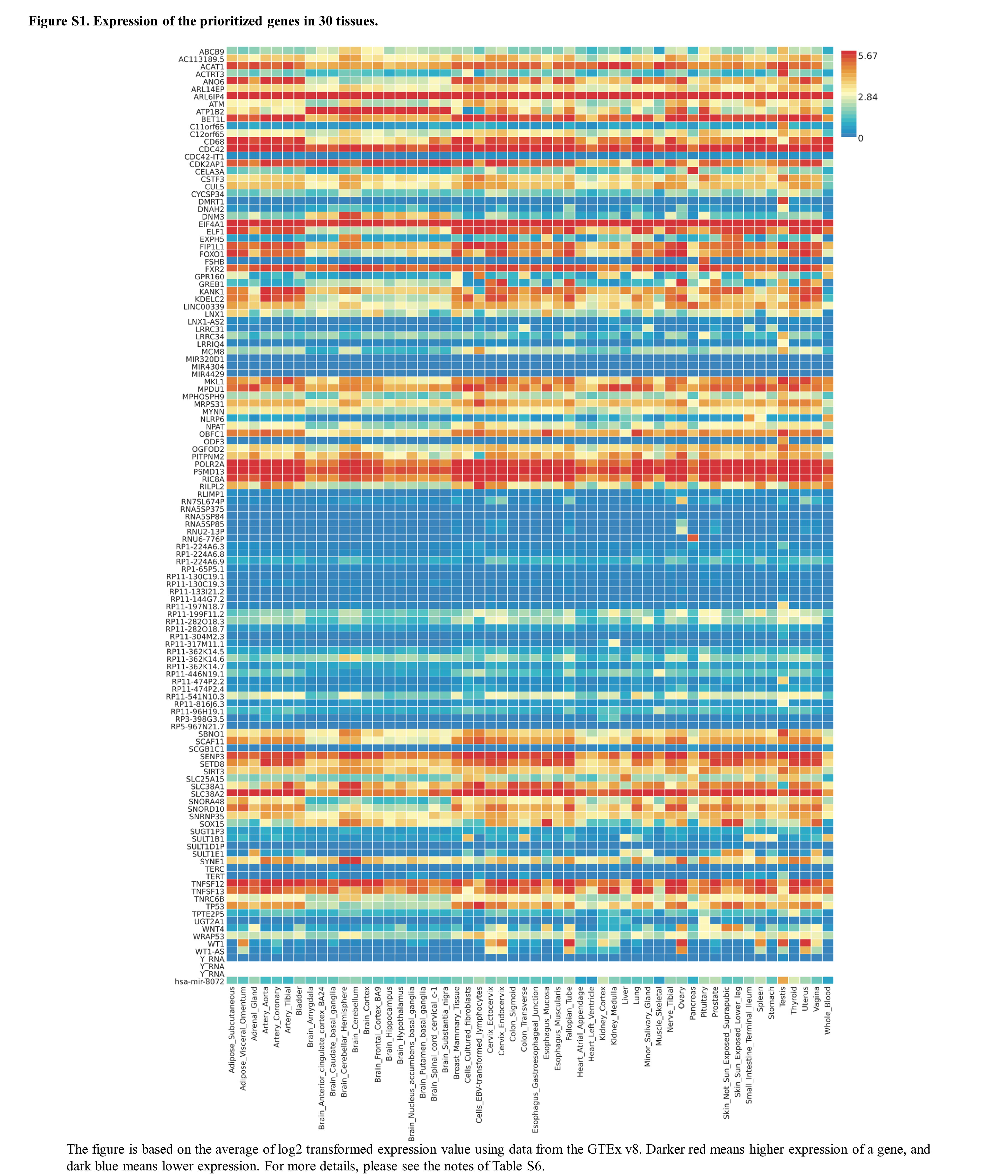

Supplement: Supplementary file 1 [file Image1.TIF]
